# Suppression of intragenic transcription requires the *MOT1* and *NC2* regulators of TATA-binding protein

**DOI:** 10.1093/nar/gkt1398

**Published:** 2014-01-22

**Authors:** Maria J. E. Koster, Asli D. Yildirim, P. Anthony Weil, Frank C. P. Holstege, H. Th. Marc Timmers

**Affiliations:** ^1^Department of Molecular Cancer Research, University Medical Center Utrecht, 3584 CG, Utrecht, The Netherlands and ^2^Department of Molecular Physiology and Biophysics, Vanderbilt University School of Medicine, Nashville, TN 37232, USA

## Abstract

Chromatin structure in transcribed regions poses a barrier for intragenic transcription. In a comprehensive study of the yeast chromatin remodelers and the *Mot1p-NC2* regulators of TATA-binding protein (TBP), we detected synthetic genetic interactions indicative of suppression of intragenic transcription. Conditional depletion of *Mot1p* or *NC2* in absence of the ISW1 remodeler, but not in the absence of other chromatin remodelers, activated the cryptic *FLO8* promoter. Likewise, conditional depletion of *Mot1p* or *NC2* in deletion backgrounds of the H3K36 methyltransferase Set2p or the Asf1p-Rtt106p histone H3-H4 chaperones, important factors involved in maintaining a repressive chromatin environment, resulted in increased intragenic *FLO8* transcripts. Activity of the cryptic *FLO8* promoter is associated with reduced H3 levels, increased TBP binding and tri-methylation of H3K4 and is independent of Spt-Ada-Gcn5-acetyltransferase function. These data reveal cooperation of negative regulation of TBP with specific chromatin regulators to inhibit intragenic transcription.

## INTRODUCTION

The repeating unit of chromatin is the nucleosome particle consisting of ∼147 bp of DNA wrapped around an octamer of histones (1). Compaction of DNA into chromatin poses a barrier to transcription, as nucleosomes compete for DNA binding with the transcription machinery and are evicted on RNA polymerase II (pol II) passage (2). Chromatin structure depends on the action of chromatin remodeling complexes, which use energy derived from ATP hydrolysis to translocate, eject or restructure nucleosomes. In the yeast *Saccharomyces cerevisiae*, these complexes are divided over four families based on their ATPase subunit: ISWI (ISW1a, ISW1b and ISW2), INO80 (INO80, SWR1), CHD and SWI/SNF (SWI/SNF and RSC) (3).

Structural integrity of chromatin is important for transcriptional fidelity, as disruption can lead to the production of transcripts from within gene bodies (4–12). Initial observations were made with mutants of the Spt6p and Spt16p (subunit of the FACT complex) histone chaperones, which revealed generation of transcripts from a cryptic promoter localized in the open reading frame (ORF) of the *FLO8* gene (10,11). Deletion of the *ISW1* and *CHD1* genes, encoding chromatin remodelers acting to position nucleosomes in ORFs, shifts intragenic nucleosomes to energetically preferred positions (6,13,14). The integrity of the repressive chromatin is also maintained by the histone H3K36 methyltransferase Set2p, which recruits the Rpd3S histone deacetylase to remove transcription elongation-associated acetylation (7,9). Likewise, alterations in transcription-dependent H3–H4 deposition by mutating factors in the HIR/Asf1p/Rtt106p pathway (5,8,12) also result in spurious intragenic transcripts.

Pre-initiation complex (PIC) formation starts with recruitment of the TATA-binding protein (TBP) (15). The assembly of pol II PICs is mainly restricted to promoters localized in nucleosome-depleted regions and is excluded from coding regions (16). Interestingly, a significant part of PICs in yeast (∼30%) is associated with non-coding RNAs (16). TBP can be recruited to promoters as part of the transcription factor IID (TFIID) complex, which consists of TBP and 13–14 TBP-associated factors (TAFs) (17), or by the Spt-Ada-Gcn5-acetyltransferase (SAGA) complex via the Spt8p/Spt3p module (18). TBP promoter occupancy is subjected to negative regulation by the Snf2/Swi2-like ATPase *Mot1p* and the negative cofactor 2 (*NC2*) complex (19,20). *In vitro, Mot1p* dissociates TBP–TATA complexes on ATP hydrolysis (21,22). *NC2* represses transcription by competing with transcription factor IIA (TFIIA) and transcription factor IIB (TFIIB) for TBP binding, thereby inhibiting PIC formation (20,23,24). In cells, TBP association to promoters is dynamic as a result of the action of *Mot1p* (25–28), and of *NC2* consisting of *NC2α* and *NC2β* (encoded by *BUR6* and *YDR1*, respectively). *In vivo, Mot1p* and *NC2* are concomitantly recruited to active promoters, where they form a complex with TATA-bound TBP to evict TBP from the promoter on ATP hydrolysis by *Mot1p* (28). Furthermore, *Mot1p* and *NC2* regulate the expression of a common set of target genes (18,29). Altogether, this indicates that *Mot1p* and *NC2* cooperate to restrict TBP binding and transcriptional activity.

Pol II promoters can be divided into two distinct classes based on TBP turnover rate. Genes with low TBP turnover correlate with TFIID dependence and weak TATA promoters, whereas genes with high TBP turnover correlate with SAGA dependence, canonical TATA-containing promoters and repression by *Mot1p* and *NC2* (18,30,31). *Mot1p* removes TBP from intrinsic preferred sites (TATA-containing) to allow binding of TBP to low-affinity binding sites (TATA-less) (32). Interestingly, a SAGA-related complex (lacking Spt8p) has been found in ORFs during transcription elongation and functions upstream of the Set2p-RPD3S pathway (33). SAGA is one of several chromatin complexes that interact with *Mot1p* (34,35).

Here, we performed a comprehensive genetic analysis to investigate interplay of the TBP regulators, *Mot1p* and *NC2*, with regulators of chromatin structure. We made use of the anchor-away (AA) technique developed by Laemmli and colleagues (36), allowing the study of essential proteins like *Mot1p* and *NC2* via conditional depletion from the nucleus. Depletion strains for *Mot1p*, *NC2α* or *NC2β* were combined with deletion or depletion alleles of chromatin-remodeling and nucleosome deposition genes. We show that a subset of these genes interacts with *Mot1p* and *NC2*. Interestingly, altering TBP function in mutants with disrupted chromatin leads to spurious intragenic (or cryptic) transcription at specific loci. Further, chromatin immunoprecipitation (ChIP) analysis revealed a cooperative control mechanism of the Isw1p chromatin remodeler and Asf1p histone chaperone with the negative TBP regulators in maintaining a repressive barrier for intragenic transcription by restricting TBP binding to the cryptic FLO8 promoter.

## MATERIALS AND METHODS

### Yeast genetics, media, plasmids and primers

All *S. cerevisiae* strains used in this study are listed in Supplementary Table S1. They were derived from HHY168 (Euroscarf #Y40343). Cells were grown in yeast extract peptone dextrose (YPD) or synthetic complete medium supplemented with 2% glucose at the indicated temperature. To create strains with a gene deletion or to C-teriminally fuse FKBP12-rapamycin-binding (FRB) domain of human mTOR to a protein of interest homologous recombination using polymerase chain reaction (PCR) generated DNA fragments was performed and verified by PCR. Details of primers and plasmids used are listed in Supplementary Tables S2 and S3, respectively. To generate the catalytic dead mutant *isw1K227R*, a linearized pRS406 plasmid containing a fragment of an ISW1 catalytically inactive mutation (a gift from Toshio Tsukiyama) was integrated using the standard pop-in/pop-out method (37) at the *ISW1* genomic locus.

### Cell culturing

For spot assays, overnight cultures from single colonies in YPD at 30°C were diluted to an OD_600_ of 0.15. Fivefold serial dilutions were prepared and spotted on YPD plates containing 1 μg/ml rapamycin, where indicated, and grown for 3 days at 30°C. For northern blot and ChIP analyses, overnight cultures in synthetic complete medium from single colonies were diluted to an OD_600_ of 0.15 and grown to OD_600_ of 0.6 at 30°C at 230 rpm. Cultures when indicated were switched to 39°C for 90 min in the presence or absence of rapamycin (1 μg/ml) and harvested. For liquid growth curves, cells were diluted to OD_600_ of 0.15 in YPD in 24 - or 48-well plates at 30°C in a Tecan Infinite F200 instrument under continuous shaking. OD_600_ was recorded every 10 min. Rapamycin (1 μg/ml) was added at an OD_600_ of 0.15 (or also at 0.6 for *STH1-FRB*, Supplementary Figure S3B) where indicated.

### RNA isolation and northern blotting

RNA isolation and northern blotting was carried out as described previously (38). RNA was isolated from 25-ml cultures by hot phenol extraction. Twenty micrograms of total RNA was loaded on a 1.2% agarose gel, with sodium phosphate buffer as the running buffer. RNA was transferred to a nylon membrane and cross-linked by ultraviolet irradiation. The membrane was prehybridized for 3 h at 42°C in prehyb mix containing 50% deionized formamide, 10% dextran sulfate, 1× P buffer [0.2% bovine serum albumin, 0.2% polyvinylpyrrolidone, 0.2% Ficoll-400, 50 mM Tris-HCl (pH 7.5), 0.1% pyrophosphate, 1% sodium dodecyl sulphate (SDS)], 100 mM NaCl and 0.2 mg/ml herring sperm. For overnight hybridization at 42°C, ^32^P-labeled strand-specific or double-stranded DNA probes were used. For strand-specific probes, a cold PCR template was made by amplification. For detection of the sense transcripts, a labeled single-stranded DNA probe was generated from the template by using the reverse primer in a linear PCR reaction. Double-stranded probes were generated using the RediPrime II kit (GE Healthcare Life Sciences). The membrane was washed twice with 2× SSC [300 mM NaCl, 30 mM sodium citrate dihydrate (pH 7.0)] at room temperature, once with 2× SSC + 1% SDS at 65°C, once with 1× SSC + 1% SDS at 65°C and once with 0.5× SSC + 1% SDS at 65°C. Analysis was carried out using a Storm 820 phosphorimager (GE Healthcare).

### Chromatin immunoprecipitation

ChIP was carried out as described previously (39), with minor modifications. In short, 225 ml of cultures was cross-linked with 1% formaldehyde for 20 min at RT at 50 rpm. The reaction was stopped with 300 mM glycine, and cells were collected by centrifugation. Cells were washed twice with ice-cold TBS and FA lysis buffer [50 mM Hepes-KOH (pH 7.5), 150 mM NaCl, 1 mM ethylenediaminetetraacetic acid, 1% Triton X-100, 0.1% sodium deoxycholate, 0.1% SDS] containing protease inhibitors. Cells were disrupted using a gene disruptor and sonicated (Bioruptor, Diagenode: 15 cycles, 30 s on/off, high setting) to produce an average fragment length of 100–300 bp. Two hundred microliters of extract was incubated overnight at 4°C with antibody [5 μg affinity-purified α-TBP, 2 μg α-H3 (Abcam ab1791) or 1 μg α-H3K4me3 (Abcam ab8580)]. Forty microliters of protein A+G beads (50% slurry) (Santa Cruz) was added and incubated for 1.5 h at 4°C. Beads were washed twice with FA-lysis buffer; twice with FA-lysis buffer containing 410 mM NaCl; twice with 10 mM Tris-HCl (pH 8), 50 mM LiCl, 1% Nonidet P-40, 1% sodium deoxycholate, 1 mM ethylenediaminetetraacetic acid and once with TE (pH 8). Samples were eluted twice with 50 μl TE-SDS 1% for 10 min at 65°C. Cross-linking was reversed overnight at 65°C with 0.1 μg/μl RNAse. Samples were treated with 2.67 μg/μl proteinase K for 2 h at 37°C, and DNA was purified using a PCR purification kit (Qiagen). Samples were analyzed by quantitative PCR, and ChIP signals were normalized relative to HMR (silent mating-type locus) signals. Experiments were repeated at least twice, but in most cases, thrice.

## RESULTS AND DISCUSSION

### Genetic interactions of *Mot1p-NC2* with specific chromatin regulators

In our previous studies of *Mot1p* and *NC2β* (18), we used the AA system (36), which relies on cytoplasmic sequestering of FRB-tagged proteins by addition of rapamycin. Nuclear depletion of *Mot1p* and *NC2β* is rapid and reduces growth of *MOT1-FRB* and *NC2β-FRB* strains on plates and in liquid cultures (18). In contrast to full-gene deletions, strains containing *MOT1-FRB* or *NC2β-FRB* conditional alleles can still form colonies on plates, permitting genetic screening (18). To allow a comprehensive study, we now succeeded in creating a *NC2α-FRB* strain, which behaves similar to the *NC2β-FRB* strain (see later in the text).

**Figure 1.**
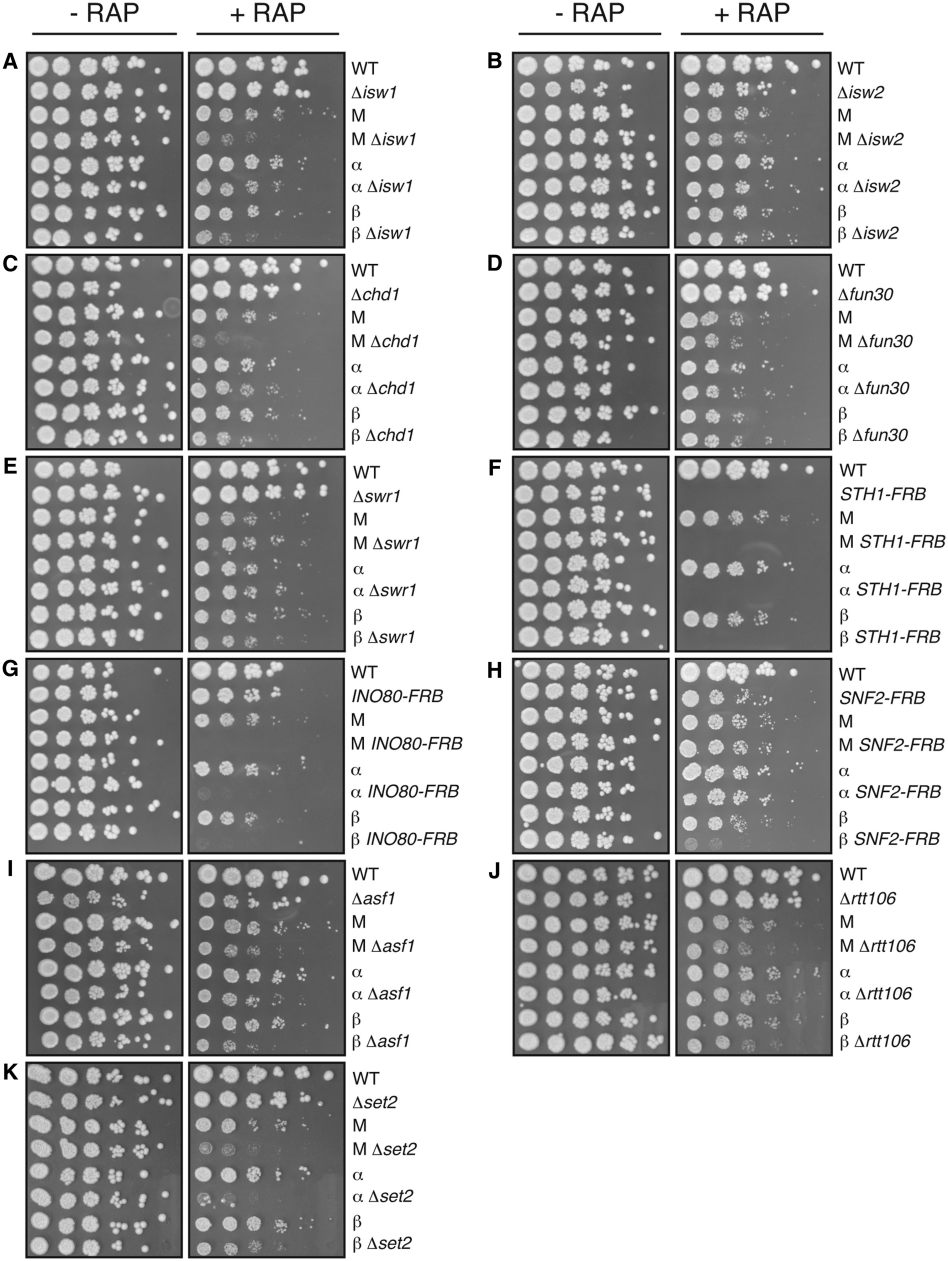
*MOT1-FRB*, *NC2α-FRB* and *NC2β-FRB* alleles display genetic interactions with several chromatin-remodeling genes. (**A–K**) Fivefold serial dilutions were spotted on YPD plates containing 1 μg/ml rapamycin and grown for 3 days at 30°C. Comparing the double AA *SNF2-FRB* strains with single AA strains (H and Supplementary Figure S2B) indicates that simultaneous depletion of two FRB-tagged proteins did not cause general abnormalities. M, *Mot1-FRB*; α, *NC2α-FRB*; *NC2β-FRB*; R, rapamycin; wt, wild-type.

To investigate interplay of *Mot1p* and *NC2* with chromatin structure regulators, the *MOT1-FRB*, *NC2α-FRB* and *NC2β-FRB* alleles were combined with deletions of the catalytic subunits of all non-essential chromatin remodelers (Δ*chd1*, Δ*fun30*, Δ*isw1*, Δ*isw2* and Δ*swr1*), the H3/H4 chaperones (Δ*asf1* and Δ*rtt106*) and the H3K36 methyltransferase (Δ*set2*). We exploited the AA technique further by creating strains that could be depleted for two essential nuclear proteins at once, by creating FRB-tagged alleles of *INO80*, *SNF2* or *STH1*. Genetic interactions were tested by analyzing growth on plates (Figure 1) or in suspension cultures in the presence of rapamycin (Supplementary Figures S1–S3). This revealed interactions with the chromatin remodelers *ISW1* (Figure 1A and Supplementary Figure S1A) and *CHD1* (Figure 1C and Supplementary Figure S1C) and a strong interaction with *INO80* (Figure 1G and Supplementary Figure S2A). No genetic interactions were observed with *ISW2* (Figure 1B and Supplementary Figure S1B), *FUN30* (Figure 1D and Supplementary Figure S1D), *SWR1* (Figure 1E and Supplementary Figure S1E) or *SNF2* (Figure 1H and Supplementary Figure S2B). As expected (40), nuclear depletion of Sth1p resulted in a rapid cell cycle arrest, which precludes growth on plates (Figure 1F). By comparing single AA strains with double AA strains in suspension growth (Supplementary Figure S3), no synthetic growth effects of *STH1* with *MOT1*, *NC2α* or *NC2β* were apparent. In conclusion, depletion of *Mot1p*, *NC2α* or *NC2β* results in synthetic growth phenotypes with depletion of Isw1p, Chd1p and Ino80p.

The INO80 complex catalyzes exchange of H2A.Z for canonical H2A relevant for maintenance of the +1 nucleosome and functions in DNA repair and replication (41,42). *Mot1p* interacts physically with the INO80 remodeler as well as with the ISW1 complex (34,35). The ISW1 and CHD1 remodelers maintain a regular chromatin structure of transcribed regions and prevent histone exchange during transcription elongation (6,13,14). In addition, the functions of Isw1p and Chd1p are linked with Spt6p and with Set2p-RPD3S to suppress intragenic transcription and with the HIR/Asf1p/Rtt106p pathways for H3–H4 deposition (4,6,10). Interestingly, the Hpc2p and Hir2p proteins of the HIR complex interact with *Mot1p* (34).

To investigate a role for *Mot1p-NC2* in intragenic transcription, we tested genetic interactions with deletions of the *ASF1*, *RTT106* or *SET2* genes. As shown in Figure 1I–K and Supplementary Figure S2C and D, the *MOT1*-, *NC2α-* and *NC2β-FRB* alleles display clear genetic interactions with the Δ*asf1* and Δ*set2* alleles and weak interactions with Δ*rtt106*. Together, the synthetic growth phenotypes suggest a role for *Mot1p* and *NC2* in suppressing intragenic transcription.

### Chromatin regulators cooperate with *Mot1p-NC2* to repress intragenic transcription

**Figure 2.**
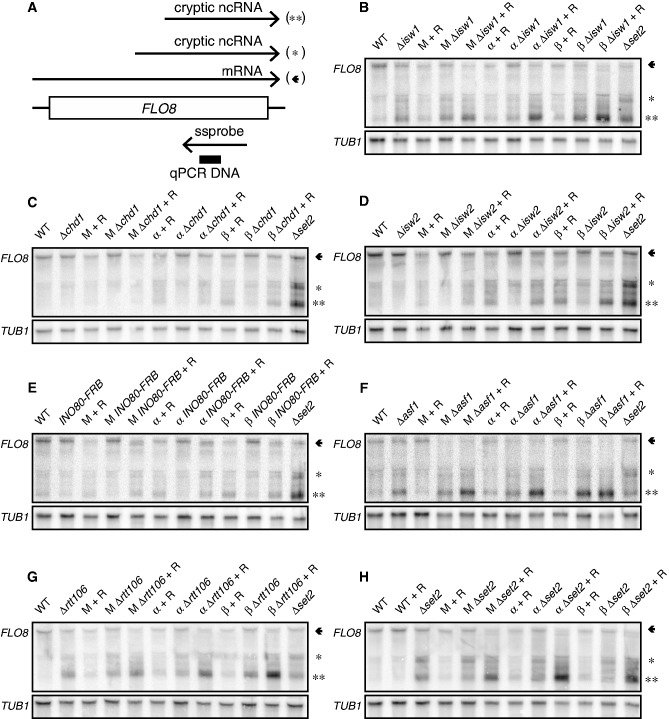
*Mot1p*, *NC2α* and *NC2β* (cooperate with Asf1p, Isw1p, Rtt106p and Set2p to prevent intragenic *FLO8* transcription. (**A**) Representation of the *FLO8* gene and its transcripts. The position of the strand-specific probe is shown, and the black box indicates the DNA fragment analyzed in ChIP. (**B–H**) Total RNA was isolated and used for northern blot analysis. Cells were grown to OD600 of 0.6 at 30°C and switched to 39°C in the presence of 1 μg/ml rapamycin, as indicated. A strand-specific DNA probe was used to detect the sense transcripts of *FLO8* and *TUB1*. The arrow and asterisks indicate the full-length and intragenic transcripts, respectively. M, *Mot1-FRB*; *α*, *NC2α*-*FRB*; *NC2β*-*FRB*; R, rapamycin; wt, wild-type.

**Figure 3.**
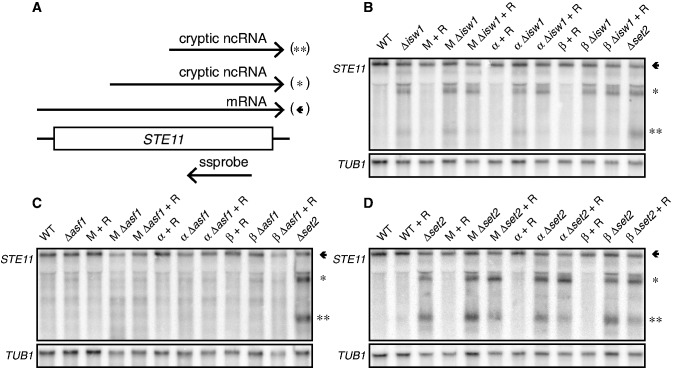
Depletion of *Mot1p*, *NC2α* and *NC2β* does not lead to intragenic *STE11* transcripts. (**A**) Representation of *STE11*, its transcripts and the strand-specific probe. (**B–D**) As in Figure 2B–J, except that analysis was performed for *STE11* transcripts. The arrow and asterisks indicate the full-length and intragenic transcripts, respectively. M, *Mot1-FRB*; α, *NC2α-FRB*; *NC2β-FRB*; R, rapamycin; wt, wild-type.

To test this, we monitored transcript species arising from the model genes *FLO8* and *STE11* (Figures 2 and 3) on (co-) depletion of *Mot1p*, *NC2* and chromatin regulators. As a positive control, an isogenic Δ*set2* strain was included, which is known to accumulate intragenic *FLO8* and *STE11* transcripts (7). Northern blotting was performed using single-stranded DNA probes for sense transcripts (Figures 2A and 3A) from cultures exposed to heat shock, which increases detection of intragenic transcripts (Supplementary Figure S4A, B). We detected intragenic transcripts from the well-characterized cryptic *FLO8* promoter in Δ*isw1,* Δ*asf1,* Δ*rtt106* and Δ*set2* strains (Figure 2B, F–H). Interestingly, depletion of *Mot1p* or *NC2* had little effect alone, but co-depletion in combination with Δ*isw1,* Δ*asf1,* Δ*rtt106* and Δ*set2* alleles increased formation of intragenic *FLO8* transcripts (Figure 2B, F–H). No cooperative effects were observed on Chd1p, Isw2p or Ino80-FRBp depletion (Figure 2C–E). Similarly, previous analyses failed to detect intragenic transcripts from the *FLO8* locus in Δ*isw2* (43) or Δ*chd1* (4,6) strains, and *INO80* was not isolated in intragenic initiation screens (4,5). In the analysis of *STE11* transcripts, we did not detect cooperative actions of *Mot1p* or *NC2* with chromatin regulators (Figure 3B–D). In fact, depletion of *Mot1p* or *NC2* in the Δ*set2* strain led to a partial reduction of intragenic *STE11* RNAs (Figure 3D).

In short, *FLO8* RNA analyses revealed cooperative actions of *Mot1p-NC2* with specific regulators (Isw1p, Asf1p, Set2p and Rtt106p) of structural integrity of chromatin in gene bodies. The suppressive effects of *Mot1p* and *NC2* on intragenic transcription are gene-specific, as no effect on *STE11* transcription was observed.

### Intragenic *FLO8* transcription is independent of SAGA and Chd1p function

**Figure 4.**
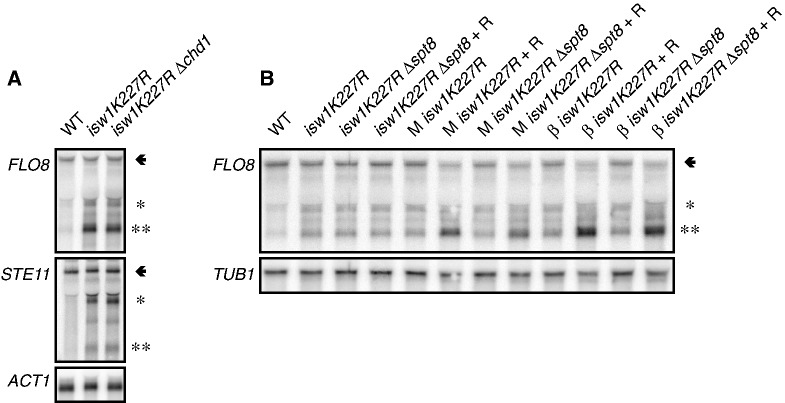
The activity of the cryptic *FLO8* promoter is not dependent on TBP recruitment by SAGA. (**A**) As in Figure 2B–J, except that double-stranded DNA probes were used, and *ACT1* messenger RNA was used as a loading control. (**B**) As in Figure 2B–J. The arrow and asterisks indicate the full-length and intragenic transcripts, respectively. M, *Mot1-FRB*; α, *NC2α-FRB*; *NC2β-FRB*; R, rapamycin; wt, wild-type.

Our observations suggest a model in which the ISW1 complex actively maintains chromatin structures to prevent exposure of cryptic promoters within genes like *FLO8*. In the presence of *Mot1p* and *NC2*, cryptic promoter activity is repressed by clearance of TBP. Analysis of the ATPase-deficient *isw1K227R* mutant allele confirmed that the enzymatic activity of the ISW1 remodelers is critical for the cooperative effects of *Mot1p* and *NC2* on intragenic *FLO8* transcripts (Figure 4B). Isw1p and Chd1p fulfill partially redundant functions, as a Δ*chd1Δisw1* mutant strain displays widespread alterations in genic nucleosome positions, spurious intragenic transcription and synthetic growth phenotypes (6,13,44). We tested whether Isw1p and Chd1p cooperate in suppressing *FLO8* intragenic transcription. However, in our *isw1K227R* strain background (W303), no elevated intragenic transcript levels were observed on deletion of *CHD1* (Figure 4A).

Cryptic promoters resemble canonical promoters in the sense that they can be SAGA- or TFIID-regulated. The *FLO8* cryptic promoter contains a functional TATA box at coordinate +1626 (4), suggesting that its activity is dependent on the SAGA complex and is repressed by *Mot1p* and *NC2* (18,30,31). Conditional depletion of *Mot1p* or *NC2* results in an increase of intragenic levels at *FLO8*. Spt8p is a critical subunit of the TBP-binding module in SAGA (45) and displays genetic interactions with *MOT1* and *NC2β* (18). To test SAGA involvement in regulation of intragenic *FLO8* transcripts by Isw1p and *Mot1p/NC2*, we deleted the *SPT8* gene in the *isw1K227R* strains. Compared with controls, no effects on intragenic *FLO8* transcription were observed (Figure 4B), indicating that SAGA is not involved in the generation of *FLO8* intragenic transcripts.

### Active cryptic promoters gain characteristics of canonical promoters

**Figure 5.**
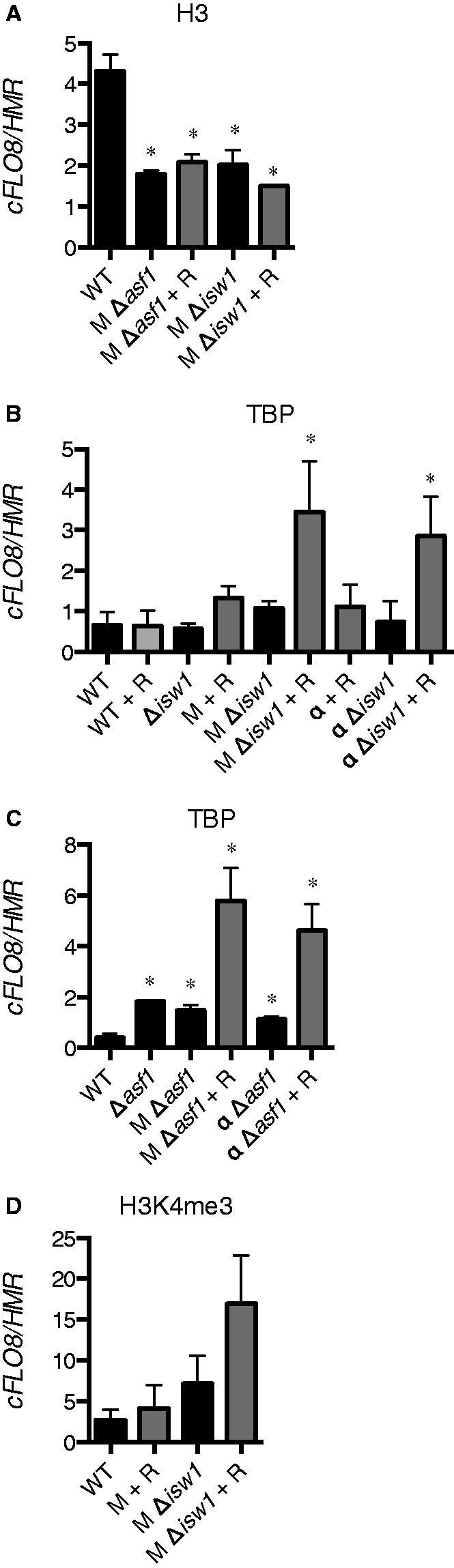
The cryptic promoter of *FLO8* (*cFLO8*) displays decreased levels of histone H3 on loss of Asf1p or Isw1p and increased TBP binding and H3K4me3 on *Mot1p* and *NC2α* depletion. (**A**) ChIP analysis of the cryptic *FLO8* promoter using H3 antibodies. Cells were grown as for RNA analysis. *cFLO8* signals were normalized relative to the silent *HMR* locus. Significant differences (*P* < 0.05 Student’s *t*-test) with the wt strain are indicated (asterisk). (**B, C**) As in (A), using TBP antibodies. (**D**) As in (A), using H3K4me3 antibodies. M, *Mot1-FRB*; α, *NC2α-FRB*; *NC2β-FRB*; R, rapamycin; wt, wild-type.

To test the model that Asf1p and Isw1p are involved in maintaining repressive chromatin at *FLO8*, we performed ChIP analysis. As expected, Δ*asf1* and Δ*isw1* cells showed loss of H3 occupancy (∼50% reduction) at the cryptic promoter of *FLO8* (Figure 5A) consistent with a more open chromatin structure. Nuclear depletion of *Mot1p* did not further reduce H3 levels (Figure 5A and Supplementary Figure S4C). This is expected, as *Mot1p* does not have chromatin as a substrate, but rather acts on TATA-bound TBP. TBP binding to the cryptic *FLO8* promoter is increased about 2-fold on nuclear depletion of *Mot1p* or *NC2α* (Figure 5B). Additional loss of Isw1p (Figure 5B) or Asf1p (Figure 5C) leads to a strong increase in TBP binding. Tri-methylation of histone H3 (H3K4me3), a hallmark of active promoters (46), increases at the cryptic *FLO8* promoter on loss of *Mot1p* and Isw1p (Figure 5D), and correlates with the appearance of intragenic *FLO8* transcripts (Figure 2B).

Our study provides novel insight into control mechanisms that ensure transcription fidelity. Previous analyses stressed the importance of histone chaperones and chromatin regulators in maintenance of a repressive chromatin conformation to prevent spurious intragenic transcription. In a genetic screen for synthetic growth phenotypes of *Mot1p* and *NC2* with chromatin regulators, we uncovered a link between TBP removal and intragenic transcription in yeast. It remains possible that intragenic transcripts have biological relevance because many of them are translated into proteins (4). Our results indicate that disruption of chromatin structure exposes a cryptic TATA-containing promoter, but this does not lead to strong intragenic transcription, as the *Mot1p* and the *NC2* complex still remove TBP from these exposed sites. However, additional loss of these negative regulators of TBP activity allows functional pol II PIC assembly at the *FLO8* cryptic promoter, resulting in formation of intragenic transcripts (Figure 2B, F, G and H). It is interesting to note that co-depletion of *Mot1p-NC2* in *ISW1* and *CHD1* deletion backgrounds displays distinct effects on *FLO8* transcription (Figure 2B and C), while these chromatin remodelers have redundant functions in maintaining intragenic nucleosome positioning (13). Possibly, nuclear depletion of *Mot1p*/*NC2α*/*NC2β* in the Δ*chd1* background affects transcription of loci other than *FLO8* or *STE11*. Interestingly, *Mot1p* has been found to interact physically with ISW1 complexes but not with the CHD1 chromatin remodeling complex (34). Isw1p is part of the ISW1a (Isw1p and Ioc3p) and ISW1b (Isw1p, Ioc2p and Ioc4p) complexes (47). Deletion of individual complex members (Ioc2p, Ioc3p or Ioc4p) did not phenocopy our Δ*isw1* results in co-suppression of intragenic *FLO8* transcription (data not shown), suggesting that both ISW1 complexes are involved.

How could the synthetic interaction between *INO80* and *MOT1/NC2α/NC2β* be explained (Figure 1G)? Inactivation of *INO80* leads to mislocalization of histone H2A.Z to transcribed regions (41). Interestingly, this histone variant was found to be involved in TBP recruitment (48,49), which could be altered by Ino80p depletion. Alternatively, *Mot1p* and *NC2* might have direct functions in DNA repair and replication, as is the case for the INO80 complex (41,42).

TATA-containing promoters are intrinsic preferred binding sites for TBP. Interestingly, previous transcriptome analysis of *spt6-1004* and *spt16-197* mutants showed that genes containing at least one TATA element [defined as TATA(A/T)(A/T)A(A/T)(A/G)] in the coding region are three times more likely to give rise to intragenic transcripts compared with genes lacking such a TATA sequence (4). *Mot1p* redistributes TBP from TATA sites to allow TBP binding to intrinsically disfavored (TATA-less) sites (32). In support of this TBP redistribution model, we observed increased TBP binding (Figure 5B and C) and transcription (Figure 2B, F, G and H, the short intragenic transcript (**)) from the TATA-containing cryptic *FLO8* promoter (+1626) (10) on nuclear depletion of *Mot1p* or *NC2*. In contrast, the long intragenic transcript arising from a location upstream of the TATA box, possibly a TATA-less promoter, remained unaffected (Figure 2B, F and G, (*)) or was reduced (Figure 2H, (*)) in expression. Likewise, the long and short transcripts arising from the *STE11* locus are differentially regulated (50). The small transcript of *STE11* arises from a TATA-less promoter (50) and is expressed in Δ*set2* (Figure 3D, the short intragenic transcript (**)). Interestingly, when *Mot1p* or *NC2* is conditionally depleted, a decrease in expression of the short intragenic transcript is observed. Again, this is consistent with a *Mot1p*/*NC2*-dependent redistribution of TBP from TATA-less to intrinsically preferred TATA-containing promoters. All together, these results stress the importance of the cooperation of *Mot1p*/*NC2* with remodelers in maintaining a repressive environment for intragenic transcription.

It is important to stress that *Mot1p*, *NC2α* and *NC2β* behave similar in our present assays. This agrees well with previous genome-wide mapping data indicating that *Mot1p* and *NC2* bind together to promoter-bound TBP, which is further substantiated by biochemical assays (18,28,51). In proteomic experiments, both *NC2* subunits were identified as *Mot1p* interactors (34). In addition, messenger RNA profiles resulting from *Mot1p* or *NC2β* depletion are similar (18,28).

Our findings have important ramifications for transcriptional fidelity of mammalian cells. Fluorescence microscopy experiments indicated that the mammalian orthologs of *Mot1p* and *NC2* collaborate to increase the off-rates of TBP from DNA in human cells (26,27). Recent genome-wide binding maps of human TBP and TFIIB revealed that the vast majority of binding events occur at non-coding transcription sites (52). We speculate that in species with larger genomes than yeast, *Mot1p* and *NC2* orthologs play an even more crucial role in suppressing spurious non-coding transcript formation.

## SUPPLEMENTARY DATA

Supplementary Data are available at NAR Online.

## FUNDING

The Netherlands Organization for Scientific Research (NWO) through ALW [820.02.013 to H.T.M.T.]; CW-TOP [700.57.302 to H.T.M.T.]; National Institutes of Health [GM52461 to P.A.W.]. Funding for open access charge: Own institution and NWO.

*Conflict of interest statement.* None declared.

## Supplementary Material

Supplementary Data
