# Supplementary material for: Suppression of intragenic transcription requires the MOT1 and NC2 regulators of TATA-binding protein
Source: Nucleic Acids Res. 2014 Jan 22;42(7):4220–9. doi: 10.1093/nar/gkt1398 (PMC3985625; doi:10.1093/nar/gkt1398)
Supplement: Supplementary Data [file supp_42_7_4220__index.html]

Suppression of intragenic transcription requires the MOT1 and NC2 regulators of TATA-binding protein — Suppression of intragenic transcription requires the MOT1 and NC2 regulators of TATA-binding protein — Supplementary Data 

# Suppression of intragenic transcription requires the *MOT1* and *NC2* regulators of TATA-binding protein

## Supplementary Data

files

**Files in this Data Supplement:**

- Supplementary Data - pdf file
