# Supplementary material for: Suppression of intragenic transcription requires the MOT1 and NC2 regulators of TATA-binding protein
Source: Nucleic Acids Res. 2014 Jan 22;42(7):4220–9. doi: 10.1093/nar/gkt1398 (PMC3985625; doi:10.1093/nar/gkt1398)

SUPPLEMENTARY DATA

SUPPLEMENTAL TABLES

Supplemental Table I.

List of yeast strains used in this study.

Name	Genotype	Source
HHY168	matalpha tor1-1 fpr1::natMX6 RPL13A-2×FKBP12::TRP1 ade2-1 leu2-3,112 HIS3-11,15 ura 3-1 can1-100 trp1-1	Euroscarf
YMK1	isogenic to HHY168 except *ade2-1::ADE2*	This study
YMK2	isogenic to YMK1 except *MOT1-FRB::HIS3*	This study
YMK3	isogenic to YMK1 except *NC2α-FRB::hphMX6*	This study
YMK4	isogenic to YMK1 except *NC2β-FRB::HIS3*	This study
YMK5	isogenic to YMK1 except *set2::kanMX6*	This study
YMK6	isogenic to YMK1 except *MOT1-FRB::HIS3 set2::kanMX6*	This study
YMK7	isogenic to YMK1 except *NC2α-FRB::HIS3 set2::kanMX6*	This study
YMK8	isogenic to YMK1 except *NC2β-FRB::HIS3 set2::kanMX6*	This study
YMK9	isogenic to YMK1 except *isw1::kanMX6*	This study
YMK10	isogenic to YMK1 except *MOT1-FRB::HIS3 isw1::kanMX6*	This study
YMK11	isogenic to YMK1 except *NC2α-FRB::hphMX6 isw1::kanMX6*	This study
YMK12	isogenic to YMK1 except *NC2β-FRB::HIS3 isw1::kanMX6*	This study
YMK13	isogenic to YMK1 except *SNF2-FRB::hphMX6*	This study
YMK14	isogenic to YMK1 except *MOT1-FRB::HIS3 SNF2-FRB::hphMX6*	This study
YMK15	isogenic to YMK1 except *NC2α-FRB::HIS3 SNF2-FRB::hphMX6*	This study
YMK16	isogenic to YMK1 except *NC2β-FRB::HIS3 SNF2-FRB::hphMX6*	This study
YMK17	isogenic to YMK1 except *INO80-FRB::hphMX6*	This study
YMK18	isogenic to YMK1 except *MOT1-FRB::HI3 INO80-FRB::hphMX6*	This study
YMK19	isogenic to YMK1 except *NC2α-FRB::HIS3 INO80-FRB::hphMX6*	This study
YMK20	isogenic to YMK1 except *NC2β-FRB::HIS3 INO80-FRB::hphMX6*	This study
YMK21	isogenic to YMK1 except *fun30::kanMX6*	This study
YMK22	isogenic to YMK1 except *MOT1-FRB::HIS3 fun30::kanMX6*	This study
YMK23	isogenic to YMK1 except *NC2α-FRB::HIS3 fun30::kanMX6*	This study
YMK24	isogenic to YMK1 except *NC2β-FRB::HIS3 fun30::kanMX6*	This study
YMK25	isogenic to YMK1 except *isw2::kanMX6*	This study
YMK26	isogenic to YMK1 except *MOT1-FRB::HIS3 isw2::kanMX6*	This study
YMK27	isogenic to YMK1 except *NC2α-FRB::hphMX6 isw2::kanMX6*	This study
YMK28	isogenic to YMK1 except *NC2β-FRB::HIS3 isw2::kanMX6*	This study
YMK29	isogenic to YMK1 except *STH1-FRB::hphMX6*	This study
YMK30	isogenic to YMK1 except *MOT1-FRB::HIS3 STH1-FRB::hphMX6*	This study
YMK31	isogenic to YMK1 except *NC2α-FRB::HIS3 STH1-FRB::hphMX6*	This study
YMK32	isogenic to YMK1 except *NC2β-FRB::HIS3 STH1-FRB::hphMX6*	This study
YMK33	isogenic to YMK1 except *chd1::kanMX6*	This study
YMK34	isogenic to YMK1 except *MOT1-FRB::HIS3 chd1::kanMX6*	This study
YMK35	isogenic to YMK1 except *NC2α-FRB::hphMX6 chd1::kanMX6*	This study
YMK36	isogenic to YMK1 except *NC2β-FRB::HIS3 chd1::kanMX6*	This study
YMK37	isogenic to YMK1 except *swr1::kanMX6*	This study
YMK38	isogenic to YMK1 except *MOT1-FRB::HIS3 swr1::kanMX6*	This study

YMK39	isogenic to YMK1 except *NC2α-FRB::HIS3 swr1::kanMX6*	This study
YMK40	isogenic to YMK1 except *NC2β-FRB::HIS3 swr1::kanMX6*	This study
YMK41	isogenic to YMK1 except *spt8::kanMX6*	This study
YMK42	isogenic to YMK1 except *isw1K227R spt8::kanMX6*	This study
YMK43	isogenic to YMK1 except *MOT1-FRB::HIS3 isw1K227R spt8::kanMX6*	This study
YMK44	isogenic to YMK1 except *NC2β-FRB::HIS3 isw1K227R spt8::kanMX6*	This study
YMK45	isogenic to YMK1 except *isw1K227R*	This study
YMK46	isogenic to YMK1 except *isw1K227R chd1::kanMX6*	This study
YMK47	isogenic to YMK1 except *rtt106::kanMX6*	This study
YMK48	isogenic to YMK1 except *MOT1-FRB::HIS3 rtt106::kanMX6*	This study
YMK49	isogenic to YMK1 except *NC2α-FRB::hphMX6 rtt106::kanMX6*	This study
YMK50	isogenic to YMK1 except *NC2β-FRB::HIS3 rtt106::kanMX6*	This study
YMK51	isogenic to YMK1 except *asf1::kanMX6*	This study
YMK52	isogenic to YMK1 except *MOT1-FRB::HIS3 asf1::kanMX6*	This study
YMK53	isogenic to YMK1 except *NC2α-FRB::hphMX6 asf1::kanMX6*	This study
YMK54	isogenic to YMK1 except *NC2β-FRB::HIS3 asf1::kanMX6*	This study

Supplemental Table II.

List of oligonucleotides used in this study.

Name	Sequence	Description
KanMX6 R	CACTCGCATCAACCAAACCGTTATTC	KAN check
HygroMX6 R	GCTCCAGTCAATGACCGCTGTTATG	HYGRO check
FRB R	TGCACCACTCTTGGGCCTCCA	FRB check
ISW1 ATPase F	AGACTGAACACGAAGAAGATGCGG	ATP isw1
ISW1 ATPase R	TAAAGGCGTTTACGTCTGGCGTCC	ATP isw1
ADE2 F	CTCTCTTCTAAGTACATCCTAC	ADE2
ADE2 R	GATGTAATCATAACAAAGCC	ADE2
STE11 F	GGATGTCACCAGAGGTGGTC	probe northern
STE11 R	GGGCACTAGGCCTGTATTGATAATC	probe northern
FLO8 F1	GCATCATCCCCATTAAGCATAGC	probe northern/ qPCR primer Flo8 cryptic
FLO8 R1	AAACCCATGTGGCGGCTTTA	probe northern/ qPCR primer Flo8 cryptic
TUB1 F	GTCCAACTGGTTTCAAGATCGGTA	probe northern
TUB1 R	CAGCAATGGATGTGGTATTTGACA	probe northern
ACT1 F	GCTGCTTTGGTTATTGATAACGG	probe northern
ACT1 R	CCAAGATAGAACCACCAATCCAG	probe northern
ISW1 F1	GGCTGTAGAGATCCAAATTCCGCAG	del isw1
ISW1 F2	GCCGTGAACTGTACAGATAGTGTTC	del isw1
ISW1 R1	CGGCTTCACGCCGCAGTATAAAG	del isw1
ISW2 F1	GGTGGTCCCTTAGACACAGCT	del isw2
ISW2 F2	CTCACTAAAAGTAACATACAGTACCG	del isw2
ISW2 R1	GTCTCTGGGATAGCACTTCACCTATC	del isw2
SPT8 F1	CTATTGTGCCACTTATACGAGCCTG	del spt8
SPT8 F2	GCAACTAAACGACCGAAATAATGC	del spt8
SPT8 R	CAGGCAACTAACTCGTCACTAGAC	del spt8
CHD1 F1	GTTCTTTCCTGATATCATTACTG	del chd1
CHD1 F2	GCAACACTTGTCGAACAGATCATAG	del chd1
CHD1 R	CAGATATTAGGTGGTGGTACCAGG	del chd1
SET2 F1	CCTATGTCAGTCCAATACCTGTTGG	del set2
SET2 F2	CATAAAGTATTAGTTTCGACATGCG	del set2
SET2 R	GTTTTATGCTCAATACTTGATGC	del set2
SWR1 F1	GATGCAGTAGTGAACATATTGC	del swr1
SWR1 F2	GTTACCCGTTTATACCTCTATACG	del swr1
SWR1 R	GAATCTTGCTCTTAAAACTCTTTG	del swr1
FUN30 F1	CATTATCAAGGCTCAAACGGCTG	del fun 30
FUN30 F2	GTAGGATTAAAAGAGATCATGAGCCC	del fun 30
FUN30 R	TCTCCCCAGATTAAAATTACCTAGC	del fun 30
ASF1 F1	CTTCCCCTGAATAGTGTGGGACG	del asf1
ASF1 F2	GCAGCCTTGCCTGACTTTACG	del asf1
ASF1 R1	GCAATAGGCGCAATCACACTTAAAC	del asf1
RTT106 F1	GGCTCTGACAATTTTCAAGACTGC	del rtt106
RTT106 F2	CCTGGACGAGAATAACGTTACAAG	del rtt106
RTT106 R1	CCTGGACGAGAATAACGTTACAAG	del rtt106
HMR F	ACGATCCCCGTCCAAGTTATG	qPCR primer HMR

HMR R	CTTCAAAAGGAGTCTTAATTTCCCTG	qPCR primer HMR
FLO8 F2	CGCCCACTAAAACTGCGAAT	qPCR primer Flo8 cryptic
FLO8 R2	TCGAAGCAACTCTCTGTCACTG	qPCR primer Flo8 cryptic
NC2BETA F1	GATTACACCACAATAGTGTATCTGATCCGGTTAAGTCGGAGGATTCTTCTCGGATCCCCGGGTTAATTAA	FRB tagging
NC2BETA R1	CCATGTGGTTTATTTTCGTATATAGCATTACTTTGTTTAGAAGCTTCTATGAATTCGAGCTCGTTTAAAC	FRB tagging
NC2BETA F2	CCGGCATAGAATTCATAATGATCCTG	FRB tagging
NC2BETA R2	TTAATTAACCCGGGGATCCGAGAAGAATCCTCCGACTTAACCGGATCAGATACACTATTGTGGTGTAATC	FRB tagging
NC2BETA F3	GTTTAAACGAGCTCGAATTCATAGAAGCTTCTAAACAAAGTAATGCTATATACGAAAATAAACCACATGG	FRB tagging
NC2BETA R3	GTGATGATGATATGAAGGAGACAGTG	FRB tagging
NC2BETA F4	CCCAAGGGTATGTTAGTTATA	FRB tagging
MOT1 F1	CTCAATACGAGGAGGAGTATAATTTAGACACCTTCATCAAAACTTTACGACGGATCCCCGGGTTAATTAA	FRB tagging
MOT1 R1	ATAAAACAAAAATGACCTTGTATACGCGTCATTCCAATGCAAGAATTTGTGAATTCGAGCTCGTTTAAAC	FRB tagging
MOT1 F2	GTTTAAACGAGCTCGAATTCACAAATTCTTGCATTGGAATGACGCGTATACAAGGTCATTTTTGTTTTAT	FRB tagging
MOT1 R2	CTAAACTGTTTCTAGTTTCCCTTTG	FRB tagging
MOT1 F3	GATTGCCTACTGTTGACCACCAAGG	FRB tagging
MOT1 R3	TTAATTAACCCGGGGATCCGTCGTAAAGTTTTGATGAAGGTGTCTAAATTATACTCCTCCTCGTATTGAG	FRB tagging
MOT1 F4	TTAACTGGTGCAGACACCGTC	FRB tagging
NC2ALPHA F1	GACAGAATAAAGACACACTTCCCTCC	FRB tagging
NC2ALPHA R1	TTAATTAACCCGGGGATCCGGGCACTCTCTTCCTCCGGTTGTGTTTGGCCTTCTTCTACGCATAGACCTT	FRB tagging
NC2ALPHA F2	GTTTAAACGAGCTCGAATTCGCAGAACGGGGCGATGTAACTTAGTGTAAAATGAATACCACATCTATATA	FRB tagging
NC2ALPHA R2	GGAGTTACTCCGGGGATCGTATGG	FRB tagging
NC2ALPHA F3	TTAATTAACCCGGGGATCCGGGCACTCTCTTCCTCCGGTTGTGTTTGGCCTTCTTCTACGCATAGACCTT	FRB tagging
NC2ALPHA R3	TATATAGATGTGGTATTCATTTTACACTAAGTTACATCGCCCCGTTCTGCGAATTCGAGCTCGTTTAAAC	FRB tagging
NC2ALPHA F4	GTTACAACACAACTACCACCA	FRB tagging
SNF F1	CTGAATCTTTCACAGATGAAGCGGACTCGAGCATGACAGAAGCGAGTGTACGGATCCCCGGGTTAATTAA	FRB tagging
SNF R1	TGTTTGTCTACGTATAAACGAATAAGTACTTATATTGCTTTAGGAAGGTAGAATTCGAGCTCGTTTAAAC	FRB tagging
SNF F2	CCTCTCCAAACCTTCCAAAGCA	FRB tagging
SNF R2	TTAATTAACCCGGGGATCCGTACACTCGCTTCTGTCATGCTCGAGTCCGCTTCATCTGTGAAAGATTCAG	FRB tagging
SNF2 F3	GTTTAAACGAGCTCGAATTCTACCTTCCTAAAGCAATATAAGTACTTATTCGTTTATACGTAGACAAACA	FRB tagging
SNF2 R3	CCTGTATATAATCTCCGAGTATTTCC	FRB tagging
SNF2 F4	CTGCGGCAACGAGTCCTATTG	FRB tagging
INO80 F1	GCAAAAGCATAAGTCAAGATGGAATTAAGGAAGCGGCAAGTGCATTGGCACGGATCCCCGGGTTAATTAA	FRB tagging
INO80 R1	GATAGACATTAACTCCGCTTAATGTAAATAACACAATATGAATACCTTTTGAATTCGAGCTCGTTTAAAC	FRB tagging
INO80 F2	CGTCGAGATTTGGTCCATGATTGG	FRB tagging
INO80 R2	TTAATTAACCCGGGGATCCGTGCCAATGCACTTGCCGCTTCCTTAATTCCATCTTGACTTATGCTTTTGC	FRB tagging
INO80 F3	GTTTAAACGAGCTCGAATTCAAAAGGTATTCATATTGTGTTATTTACATTAAGCGGAGTTAATGTCTATC	FRB tagging
INO80 R3	CACCCGTTTATGACGTCGATCC	FRB tagging
INO80 F4	GGATGGTTCCTCAAAGTTGGAGG	FRB tagging
STH1 F1	CTGACAAGTTAAATGAGTTTACTGATGAATGGTTCAAGGAACACTCTTCGCGGATCCCCGGGTTAATTAA	FRB tagging
STH1 R1	GGGGAAAGGGATATAGTCGTAAAAAAAAAAAACATGTGGTGATGAAAACGGAATTTGTGAATTCGAGCTCGTTTAAAC	FRB tagging

STH1 F2	CCAGAGAGTAAATCGCCCGC	FRB tagging
STH1 R2	GGGGAAAGGGATATAGTCGTAAAAAAAAAAAACATGTGG TGATGAAAACGGAATTTGTGAATTCGAGCTCGTTTAAAC	FRB tagging
STH1 F3	GTTTAAACGAGCTCGAATTCACAAATTCCGTTTTCATCAC CACATGTTTTTTTTTTTTACGACTATATCCCTTTCCCC	FRB tagging
STH1 R3	CCGCAGTCCCTTTGCCAATC	FRB tagging
STH1 F4	CCGAAACTGACCGTCAAGATCAAAC	FRB tagging
SNF2 F1	CTGAATCTTTCACAGATGAAGCGGACTCGAGCATGACAG AAGCGAGTGTACGGATCCCCGGGTTAATTAA	FRB tagging
SNF2 R1	TGTTTGTCTACGTATAAACGAATAAGTACTTATATTGCTTT AGGAAGGTAGAATTCGAGCTCGTTTAAAC	FRB tagging
SNF2 F2	CCTCTCCAAACCTTCCAAAGCA	FRB tagging
SNF2 R2	TTAATTAACCCGGGGATCCGTACACTCGCTTCTGTCATGC TCGAGTCCGCTTCATCTGTGAAAGATTCAG	FRB tagging
SNF2 F3	GTTTAAACGAGCTCGAATTCTACCTTCCTAAAGCAATATA AGTACTTATTCGTTTATACGTAGACAAACA	FRB tagging
SNF2 R3	CCTGTATATAATCTCCGAGTATTTCC	FRB tagging
SNF2 F4	CTGCGGCAACGAGTCCTATTG	FRB tagging

Supplemental Table III.

List of plasmids used in this study.

Plasmid	Source	Description
pRS406-ISW1-GRT	Tsukiyama	Fragment of ISW1 gene with catalytically inactive mutation
pfa6a-HphNT1	Euroscarf	
pfa6a-FRB-his3MX6	Euroscarf	
pfa6a-FRB-hphMX6	This study	

SUPPLEMENTAL FIGURE LEGENDS

Supplemental Figure 1. *Mot1-FRB*, *NC2α-FRB* and *NC2β-FRB* display genetic interactions with subunits of several chromatin-remodeling complexes. (**A-E**) Cells were diluted to OD_{600} of 0.15 and grown for 2 days at 30°C in the absence or presence of 1 µg/ml rapamycin as indicated under continuous shaking. OD_{600} was measured every 10 minutes and displayed on the y-axis as $\log_2$. M, *Mot1-FRB*; α, *NC2α-FRB* β; *NC2β-FRB*; R, rapamycin; wt, wild-type.

Supplemental Figure 2. *Mot1-FRB*, *NC2α-FRB* and *NC2β-FRB* display genetic interactions with subunits of several chromatin-remodeling complexes. (**A-E**) As in Fig. S1. M, *Mot1-FRB*; α, *NC2α-FRB* β; *NC2β-FRB*; R, rapamycin; wt, wild-type.

Supplemental Figure 3. *Mot1-FRB*, *NC2α-FRB* and *NC2β-FRB* do not display genetic interactions with *STH1-FRB*. (**A**) As in Fig. S1. (**B**) As in Fig. S1, except rapamycin was added at OD_{600} of 0.6. M, *Mot1-FRB*; α, *NC2α-FRB* β; *NC2β-FRB*; R, rapamycin; wt, wild-type.

Supplemental Figure 4. Intragenic transcription from the *FLO8* gene is revealed in Δ*isw1* cells upon heat-shock. (**A**) As in Fig. 2 (panels **B-J**), except that cells were grown at 30°C, double-stranded DNA probes were used and *ACT1* mRNA was used as a loading control. (**B**) As in Fig. 2 (panels **B-J**). (**C**) As in Fig. 4 panel **A**.

Supplemental Figure 1

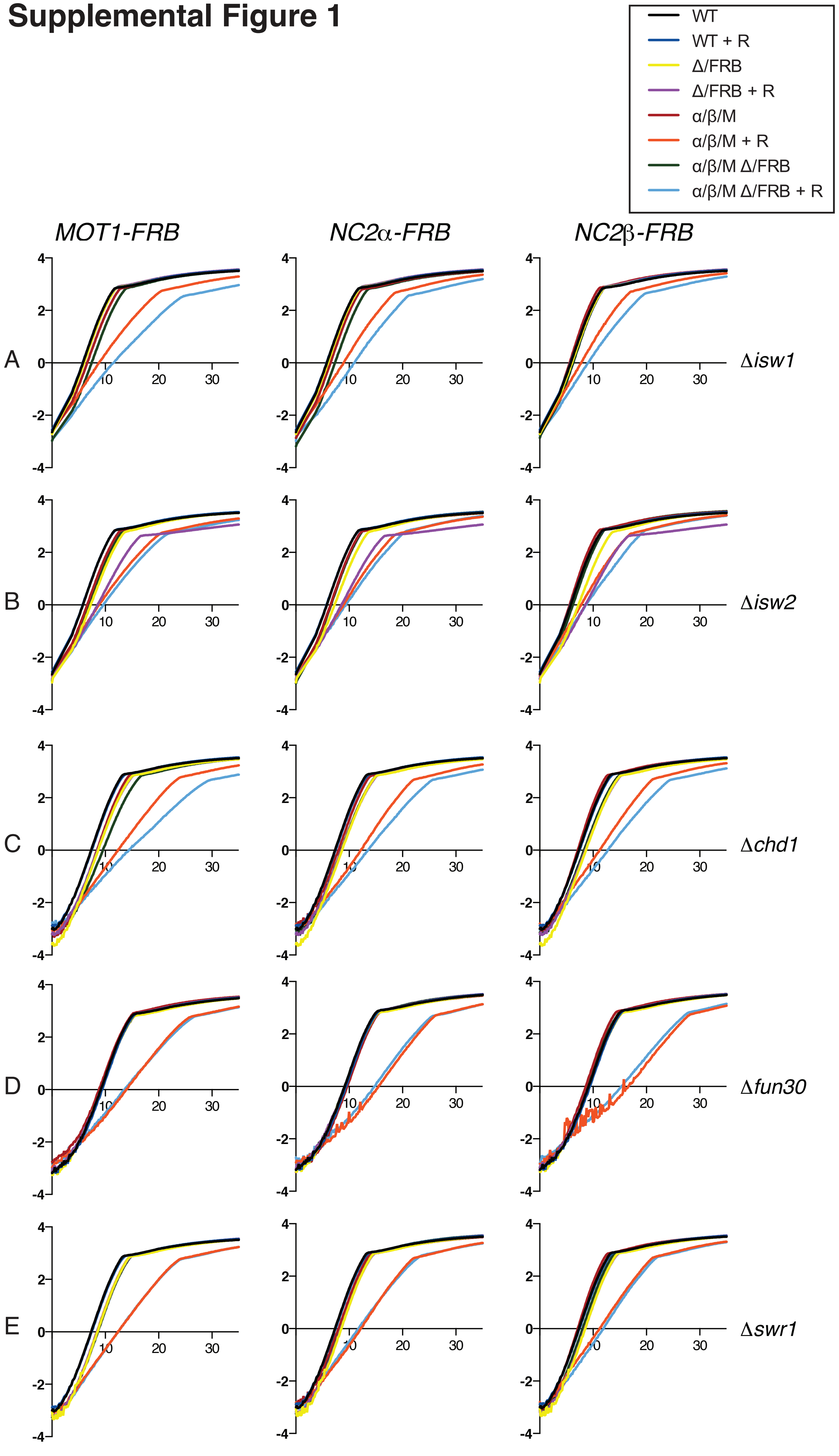

Supplemental Figure 2

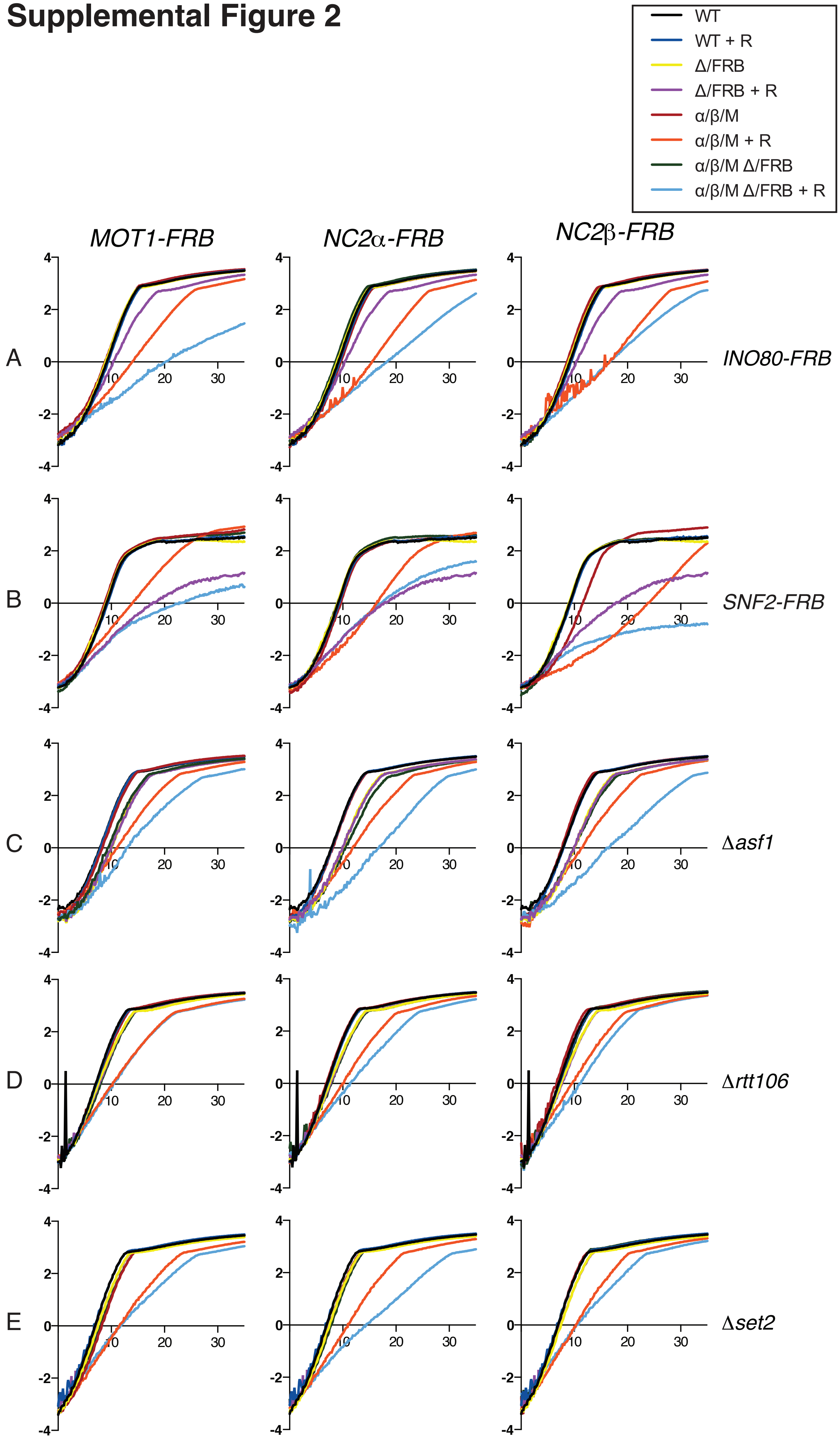

Supplemental Figure 3

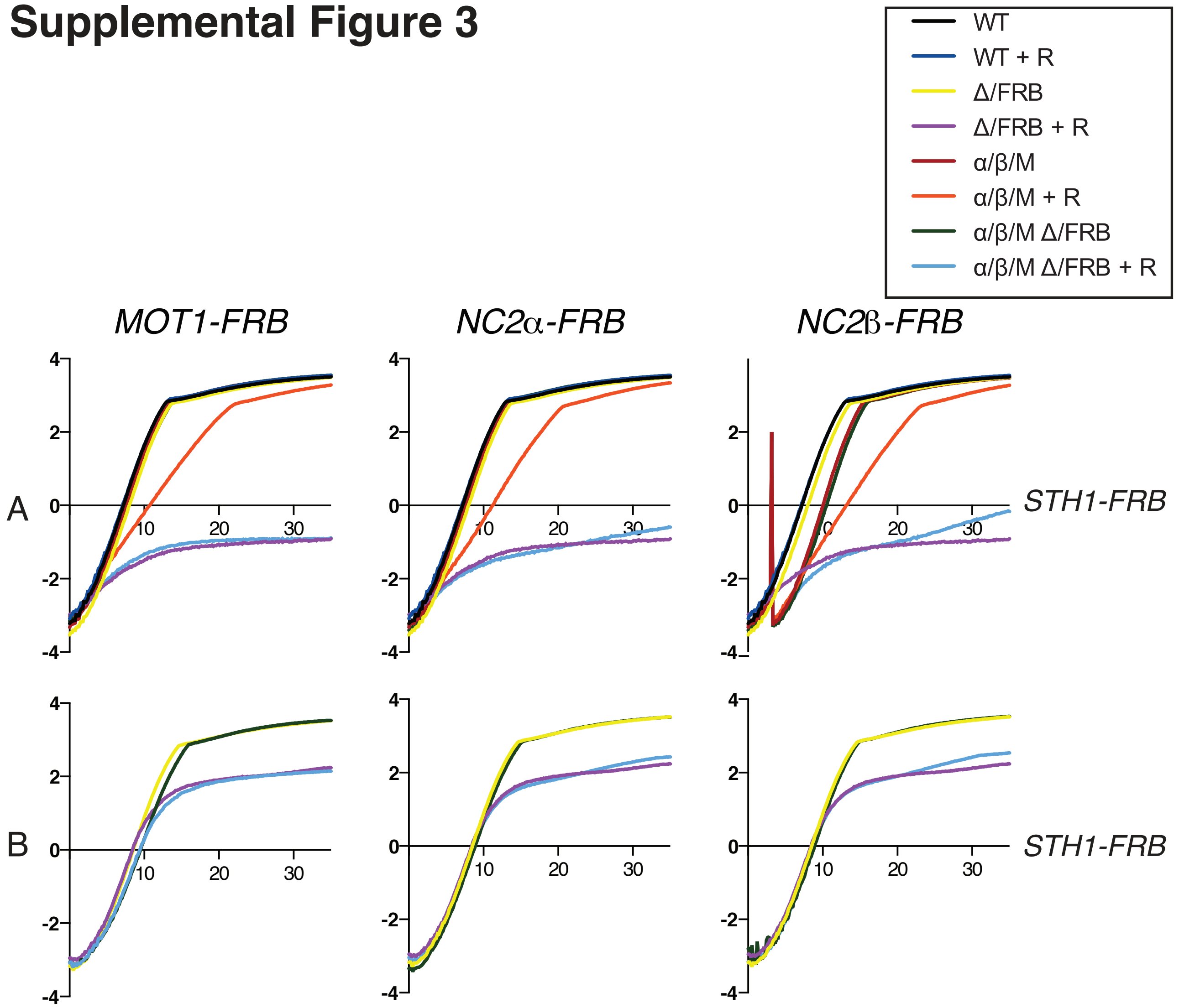

Supplemental Figure 4

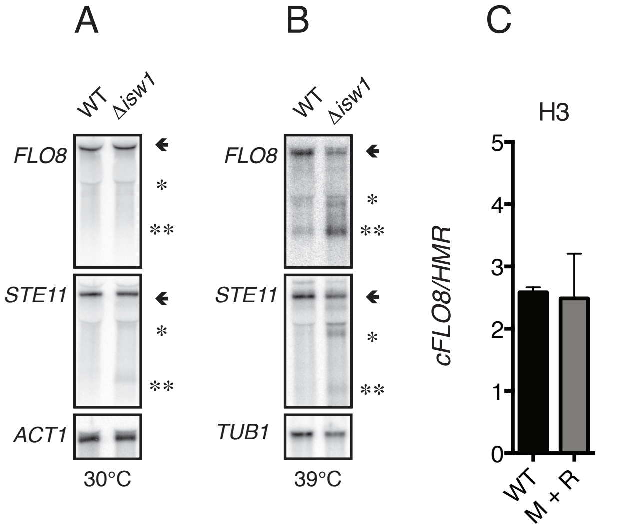

Supplement: Supplementary Data [file supp_gkt1398_nar-03372-x-2013-File002.pdf]
